# Modeling a variant of unknown significance in the *Drosophila* ortholog of the human cardiogenic gene *NKX2.5*

**DOI:** 10.1242/dmm.050059

**Published:** 2023-09-29

**Authors:** TyAnna L. Lovato, Brenna Blotz, Cayleen Bileckyj, Christopher A. Johnston, Richard M. Cripps

**Affiliations:** ^1^ Department of Biology, University of New Mexico, Albuquerque, NM 87131, USA; ^2^ Department of Biology, San Diego State University, San Diego, CA 92182, USA

**Keywords:** Heart, *Drosophila*, *NKX2.5*, *tinman*, Congenital heart disease, Variant of unknown significance

## Abstract

Sequencing of human genome samples has unearthed genetic variants for which functional testing is necessary to validate their clinical significance. We used the *Drosophila* system to analyze a variant of unknown significance in the human congenital heart disease gene *NKX2.5* (also known as *NKX2-5*). We generated an R321N allele of the *NKX2.5* ortholog *tinman* (*tin*) to model a human K158N variant and tested its function *in vitro* and *in vivo*. The R321N Tin isoform bound poorly to DNA *in vitro* and was deficient in activating a Tin-dependent enhancer in tissue culture. Mutant Tin also showed a significantly reduced interaction with a *Drosophila* T-box cardiac factor named Dorsocross1. We generated a *tin^R321N^* allele using CRISPR/Cas9, for which homozygotes were viable and had normal heart specification, but showed defects in the differentiation of the adult heart that were exacerbated by further loss of *tin* function. We propose that the human K158N variant is pathogenic through causing a deficiency in DNA binding and a reduced ability to interact with a cardiac co-factor, and that cardiac defects might arise later in development or adult life.

## Teaser

**Summary:** A variant of unknown clinical significance of a human cardiogenic gene was modeled in *Drosophila*. *In vitro* assays showed defects in protein function, and mutation *in vivo* revealed defects in heart structure.

## INTRODUCTION

Approximately 1% of children born in the USA suffer some form of congenital heart condition that can lead to significant mortality or morbidity (Hoffman and Kaplan, 2002; Reller et al., 2008). Even when these conditions are surgically repaired, the patient can suffer long-term effects upon cardiac performance and overall health. Clearly, a better understanding of the processes that are affected as the heart is developing will provide insight into mechanisms for understanding and treating congenital heart disease. As a result, there has been intense focus upon defining the regulatory network controlling heart development (Waardenberg et al., 2014).

Research over the past 30 years has determined that heart formation arises through the action of a conserved transcriptional and signaling network that promotes heart cell specification and development (for reviews, see, for example, Bodmer and Venkatesh, 1998; Cripps and Olson, 2002; Xia et al., 2020). During this time, the value of engaging a diverse group of model organisms to generate mechanistic understanding of cardiac development and disease has been exemplified by studies of the *tinman/Nkx2.5* genes. The gene *tinman* (*tin*) was first discovered in *Drosophila* (Kim and Nirenberg, 1989) and shown to be both expressed in and required for the formation of the *Drosophila* heart (Bodmer, 1993; Azpiazu and Frasch, 1993). Subsequently, the mammalian ortholog of *tin* was identified as *Nkx2.5* (also known as *Nkx2-5*) (Lints et al., 1993; Komuro and Izumo, 1993) and shown to be required for normal heart development in mice (Lyons et al., 1995). Shortly afterwards, variants in human *NKX2.5* were associated with congenital heart defects in three separate human pedigrees (Schott et al., 1998). There have since been numerous examples demonstrating that conserved processes control heart development across the animal kingdom, and that mutations in these conserved genes impact cardiogenesis across multiple organisms (McCulley and Black, 2012).

Through this research, it has become apparent that a significant proportion of instances of human congenital heart defects arise from variants in cardiac transcription factors. With the advent of whole-exome or whole-genome sequencing, a trove of natural variants in many transcription factor genes has been identified and deposited in databases such as ClinVar. Many of these variants are known to be pathogenic based upon clear association with clinical outcomes, as is the case with several *NKX2.5* variants (see, for example, Schott et al., 1998; McElhinney et al., 2003). Furthermore, studies have sought to define the functional significance of human *NKX2.5* variants using *in vitro* and tissue culture studies (Kasahara and Benson, 2004; Dixit et al., 2021), to assess the impact of mutations in the context of cardiomyocytes differentiating *in vitro* (Bouveret et al., 2015), and some disease alleles have been modeled in mice (Costa et al., 2013; Furtado et al., 2017).

More broadly, studies in the mouse system have been successful in defining the developmental impacts of a number of mutations known to cause congenital heart disease, affecting several known cardiogenic genes (Majumdar et al., 2021). Most of these studies have focused upon known pathogenic mutations, whereas mutations with unknown molecular or clinical effects have been understudied. Mutations in this latter category should be a focus of study, however, because it is important to know whether those changes require more detailed analysis to determine whether they contribute to human disease. Moreover, there is an increasing number of human variants of unknown impact (Telenti et al., 2016), and the pace of identification of new variants has outstripped the ability to functionally or practically test them in vertebrate systems to determine whether they have clinical significance.

Given the conserved nature of heart specification and development across higher animals, one approach to efficiently test the functional significance of human variants is to model them in genetically amenable animals. This allows whole-organism studies to be carried out for a particular mutation in a more high-throughput manner, and can provide insight into the potential clinical relevance of a newly discovered human variant. Here, we test this approach by modeling in *Drosophila tin* a basic [arginine (Arg) in *Drosophila*, lysine in human] to polar asparagine (Asn) missense mutation identified in *NKX2.5*, for which the clinical significance is unknown. We use biochemical tests *in vitro*, and a genome-edited *in vivo* allele, to demonstrate that this mutation attenuates protein function and results in reproducible defects in adult heart structure. We conclude that this variant is likely to be pathogenic in humans and propose that modeling human variants in *Drosophila* can be an effective way to assess the functional significance of a variant.

## RESULTS

### *In vitro* analysis of the R321N mutation

**Fig. 1.**
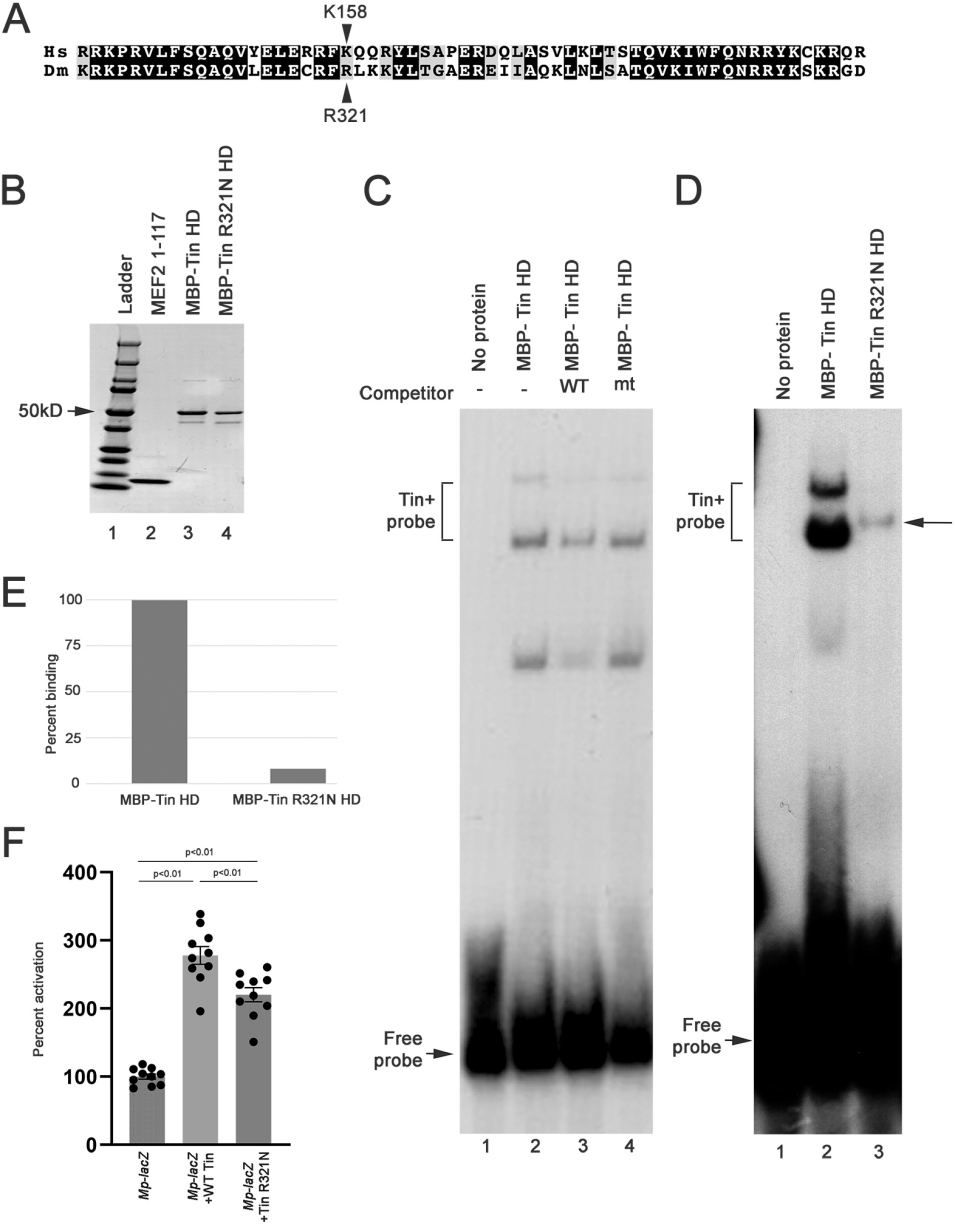
**Functional analysis of Tin^R321N^ *in vitro*.** (A) Comparison of the homeodomain sequences of human (Hs) NKX2.5 and *Drosophila melanogaster* (Dm) Tinman (Tin). The location of the point mutation being analyzed is indicated. (B) Purified native proteins used for DNA-binding assays. Equivalent amounts of wild-type (WT) MBP-Tin (lane 3) and mutated MBP-Tin (lane 4) were purified. The purified MEF2 was used for a different project. (C) Electrophoretic mobility shift assay demonstrating a specific interaction between WT Tin and the Multiplexin (Mp) Tin-binding site. In the absence of added protein, the free probe has high mobility (lane 1), but a portion of this probe has reduced mobility in the presence of WT Tin protein (lane 2). This interaction is specific, because the intensity of the bound complex is reduced in the presence of a 300-fold excess of non-radioactive Tin sequence (lane 3) but not reduced in the presence of excess non-radioactive mutant (mt) sequence (lane 4). Representative gel from duplicate experiments. (D) Electrophoretic mobility shift assay testing the ability of WT MBP-Tin homeodomain (MBP-Tin HD) and MBP-Tin^R321N^ HD to bind to a consensus Tin-binding site in the *Mp* gene. Note that a Tin+probe complex is formed in both the WT and mutant lanes (lanes 2 and 3), but that the intensity of the shifted probe is strongly reduced in the mutant, despite adding the same amount of purified protein. This electrophoretic mobility shift image is a representative image from two replicates. (E) Quantification of the band intensity from panel D. (F) Co-transfection assays assessing the ability of WT and mutant Tin to activate an enhancer-*lacZ* reporter from the *Mp* gene. Although both Tin isoforms can activate the reporter above background levels, Tin^R321N^ shows a significantly reduced activation ability compared to that of WT. Data are from ten replicates. One-way ANOVA of the three sample sets determined significant differences between the treatments (*P*<0.01), and post-hoc Tukey tests were applied to each pairwise comparison.

The K158N variant of human *NKX2.5* was submitted to ClinVar in 2017 (accession VCV000373685.1). This variant was classified to be of unknown significance and was therefore identified as a suitable allele to model in *Drosophila*, where clinical insight might be gained from analysis in a model system. In addition, residue K158 of NKX2.5 lies at the end of the first alpha helix of the homeodomain (Pradhan et al., 2012) and is one of three amino acids shown to directly contact TBX5 in a ternary structure with DNA (Luna-Zurita et al., 2016). These observations suggest that the K158N variant might contribute to human disease, but no functional testing was available to corroborate this hypothesis. In the orthologous *Drosophila* gene, *tin*, residue R321 of Tin corresponds to K158 in NKX2.5, which conserves the basic nature of the amino acid (Fig. 1A). To determine whether the NKX2.5 variant affects protein function, we modeled the basic-to-Asn mutation in *tin* and assessed its activity *in vitro* and *in vivo*.

We first generated native protein corresponding to the homeodomains of wild-type (WT) and R321N mutant Tin fused to maltose binding protein (MBP) and purified these to near homogeneity (Fig. 1B). Next, we wanted to use each protein in an electrophoretic mobility shift assay to determine whether the proteins could bind to a known Tin-binding site. We selected a site from the cardiac enhancer of the *Multiplexin* (*Mp*) gene, which encodes a collagen protein expressed in the *Drosophila* heart (Lovato et al., 2023 preprint). This site binds to WT MBP-Tin protein (Fig. 1C, lane 2), and competition of this complex by non-radioactive WT but not mutant sequence (Fig. 1C, lanes 3 and 4, respectively) indicates that the Tin-DNA interaction is specific. We next tested the ability of the Tin^R321N^ protein to bind to this site. Whereas the WT MBP-Tin homeodomain bound robustly to the radioactive probe (Fig. 1D, lane 2), an equal amount of the MBP-Tin homeodomain containing the R321N change reproducibly only bound weakly to the target sequence (Fig. 1D, lane 3), indicating that the point mutation affects protein-DNA interaction. We used densitometry to quantify the differences in binding and found that the intensity of the mutant band was 7% that of the WT, despite equal amounts of Tin protein added to each lane (Fig. 1E).

We further tested the impact of the R321N mutation in transient co-transfection experiments. Here, we transfected into *Drosophila* S2 tissue culture cells expression plasmids expressing WT or R321N-mutant full-length Tin, plus a reporter plasmid comprising an *Mp* cardiac enhancer fused to the *lacZ* gene. Transfected cells were lysed and assessed for the accumulation of β-Galactosidase (βGal) expressed from the reporter, normalized to βGal expression in the presence of empty expression plasmid. We found that WT Tin activated the reporter significantly, whereas the R321N isoform activated reporter expression more weakly. Nevertheless, the reduced activation from the mutant construct was still significantly greater than baseline activation (Fig. 1F).

Taken together, these results indicate that the R321N mutation attenuates Tin function, but that some significant activity still remains in the mutant isoform.

### Interaction of *tin* with the TBX ortholog *Dorsocross1*

The crystal structure of a complex between DNA, NKX2.5 and TBX5 was recently described (Luna-Zurita et al., 2016; Pradhan et al., 2016), and, interestingly, one of the three residues of NKX2.5 that interacts directly with TBX5 is K158. Moreover, combined mutation of K158 plus the two other interacting residues reduced the interaction between NKX2.5 and TBX5 (Luna-Zurita et al., 2016). In *Drosophila*, although no physical interaction of Tin with any TBX proteins has been described, a genetic interaction between *tin* and three *TBX6* orthologs [named *Dorsocross1-3* (*Doc1-3*)] has been documented. Specifically, loss of function for *tin* increases the severity of cardiac patterning defects arising from *Doc* mutations (Reim and Frasch, 2006). We therefore sought to determine whether this genetic interaction is also reflected at the protein level. In addition, given the strong conservation in T-box sequences between paralogs, we determined whether the strength of any interaction between Tin and a Doc protein was affected by the R321N mutation.

**Fig. 2.**
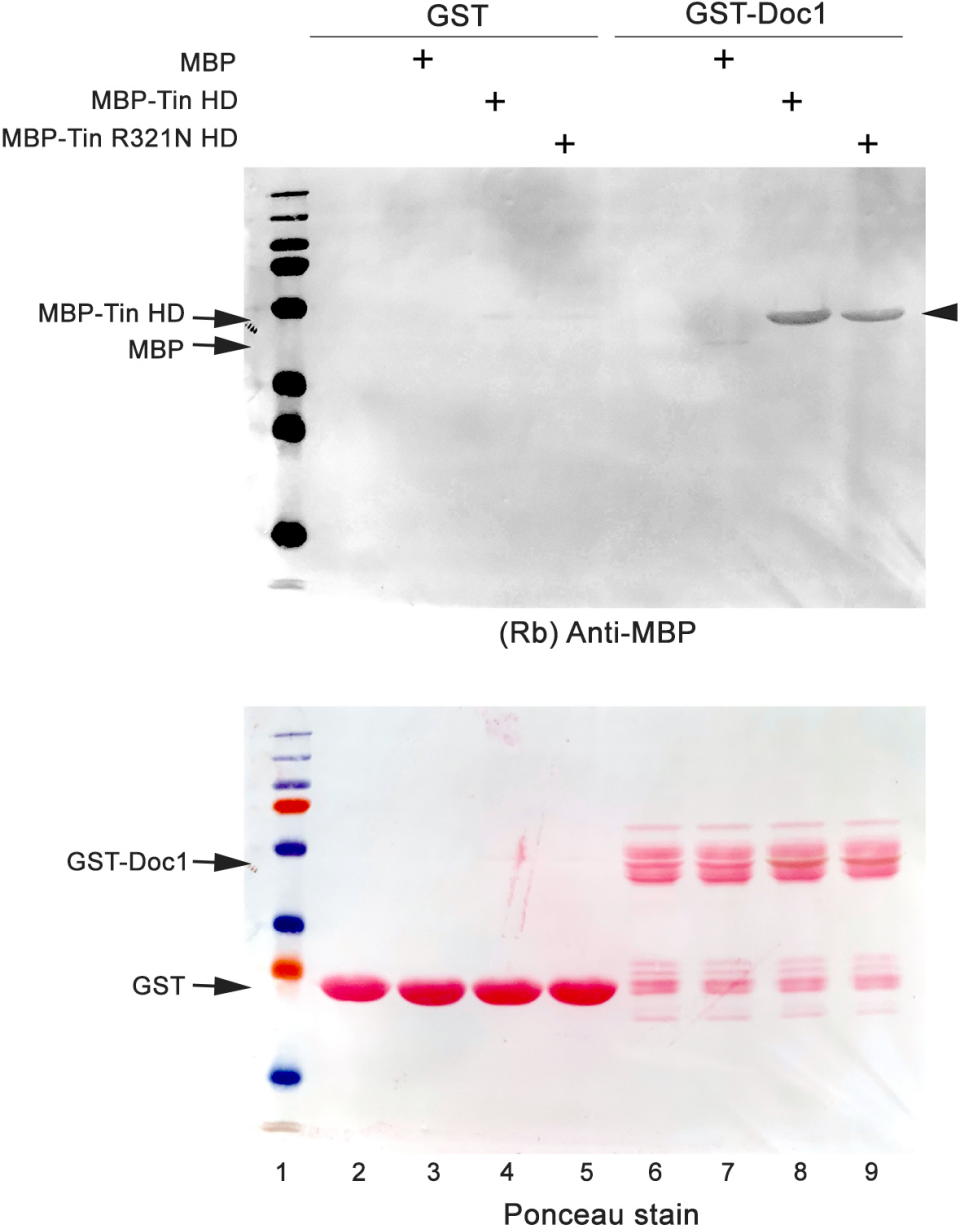
**The Tin homeodomain interacts directly with Doc1.** GST alone or fused to Doc1 (GST-Doc1) was immobilized as solid-phase bait on glutathione agarose, washed extensively, and subsequently incubated without or with 10 µM MBP alone or MBP-Tin (WT or R321N mutant) homeodomain (HD) prey proteins as indicated. Following incubation, reactions were extensively washed and resolved by SDS-PAGE. Owing to the overlapping molecular masses of GST-Doc1 and MBP-Tin, bound MBP-Tin was visualized in western blots using an anti-MBP antibody (top panel, arrowhead). Ponceau Red staining of the blot was done following western blot development to ensure equal GST and GST-Doc1 loading and transfer (bottom panel). Binding to GST was minimal for all MBP proteins. GST-Doc1 did not show a significant interaction with MBP alone but did bind both WT MBP-Tin and MBP-Tin^R321N^ at this single concentration. Binding to the MBP-Tin^R321N^ appeared reduced compared to that to WT MBP-Tin. Data for interactions with WT MBP-Tin are representative of five independent experiments. Data for interaction with MBP-Tin^R321N^ are from a single experiment, but were further investigated in Fig. 3.

We first determined whether a physical interaction between Tin and Doc could be documented. We selected Doc1 to test, because the T-box DNA-binding domain in this isoform is identical to that of Doc2, and these two isoforms are most similar to human TBX6. We performed pulldown experiments using purified MBP-Tin WT and R321N prey protein (10 µM of each) and tested their interaction with GST-Doc1 bait protein immobilized on glutathione agarose. We found that whereas MBP alone had negligible binding to either GST or GST-Doc1 (Fig. 2, lanes 3 and 7, respectively), significant binding of MBP-Tin with GST-Doc1 was evident (Fig. 2, lane 8). This binding was absent when MBP-Tin was tested with GST alone as a control (Fig. 2, lane 4). These data demonstrate, for the first time, that Tin can physically interact with a T-box protein. The data presented here demonstrating interaction of WT Tin with Doc1 are representative of five replicate experiments, and importantly demonstrate that the Tin-Doc interactions are due to the transcription factor sequences, rather than due to the presence of their GST or MBP tags.

**Fig. 3.**
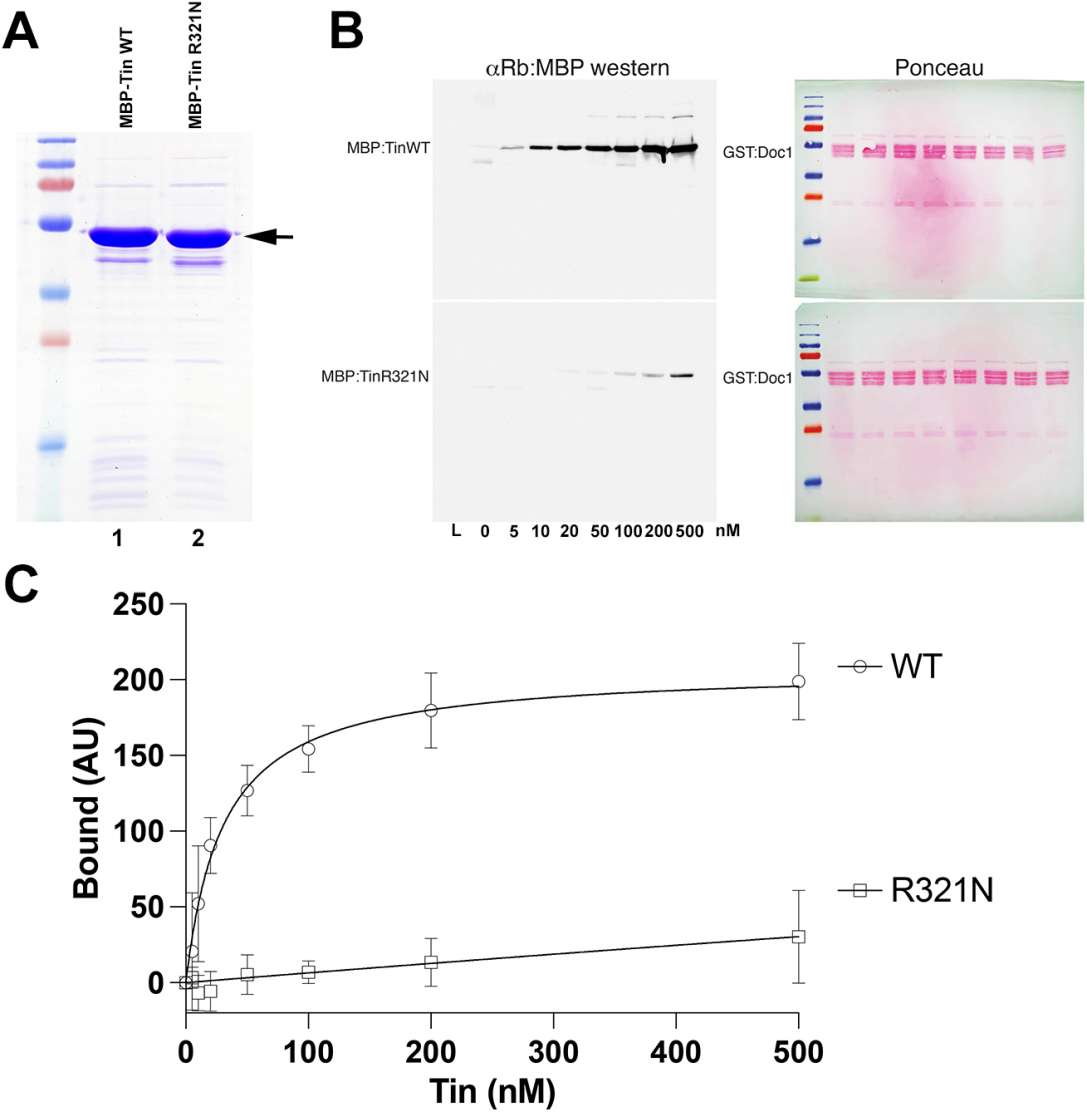
**The Tin^R321N^ isoform interacts with Doc1, but with a significantly reduced affinity.** (A) WT and R321 Tin isoforms were purified to near homogeneity (arrow); 10 µg of each protein preparation is shown. (B) Equal amounts of GST-Doc1 were immobilized on glutathione agarose, washed extensively, and subsequently incubated in the presence of increasing concentrations (0-500 nM) of WT MBP-Tin or MBP-Tin^R321N^. Following incubation, reactions were extensively washed and resolved by SDS-PAGE. Bound MBP-Tin proteins were visualized by western blot analysis using an anti-MBP antibody (left panels); GST-Doc1 was visualized using Ponceau Red staining of blots (right panels). WT MBP-Tin (top-left panel) bound robustly across the concentration range tested, whereas MBP-Tin^R321N^ (bottom-left panel) was significantly impaired in GST-Doc1 binding. (C) Saturation binding curves of the mean±s.d. from five independent experiments demonstrate the effects of R321N mutation on Doc1 binding. Bound MBP-Tin proteins were quantified in ImageJ and normalized to the total GST-Doc1 for respective samples. AU, arbitrary units.

We next tested the ability of MBP-Tin^R321N^ mutant to bind to GST-Doc1. In a single initial experiment, we found that there was interaction between GST-Doc1 and MBP-Tin^R321N^ (Fig. 2, lane 9). However, this binding appeared to be diminished compared to that of WT protein and prompted us to perform a thorough affinity-binding analysis as described below.

These initial interaction studies examined only a single concentration of MBP-Tin proteins binding to GST-Doc1. To determine the affinity of these interactions and reproducibly assess whether this interaction is affected by the R321N substitution, we next performed saturation binding experiments by examining binding across a lower range of MBP-Tin protein concentrations, using purified WT Tin and Tin^R321N^ proteins purified to near homogeneity (Fig. 3A). The WT MBP-Tin bound robustly across nanomolar concentrations (Fig. 3B) with kinetics consistent with a one-site saturation binding (Fig. 3C). In contrast, MBP-Tin^R321N^ binding was significantly impaired at these lower concentrations, revealing a severe impact of this mutation on Doc1 binding. These data demonstrate that Tin binds Doc1 with a high affinity and that the R321N mutant dramatically impairs this interaction.

Taken together, our *in vitro* and tissue culture studies indicate that the R231N mutation significantly impacts several aspects of Tin function, including DNA binding, transactivation of target genes and interaction with an essential cardiac co-factor.

### Generation and analysis of a *tin^R321N^* mutant *in vivo*

**Fig. 4.**
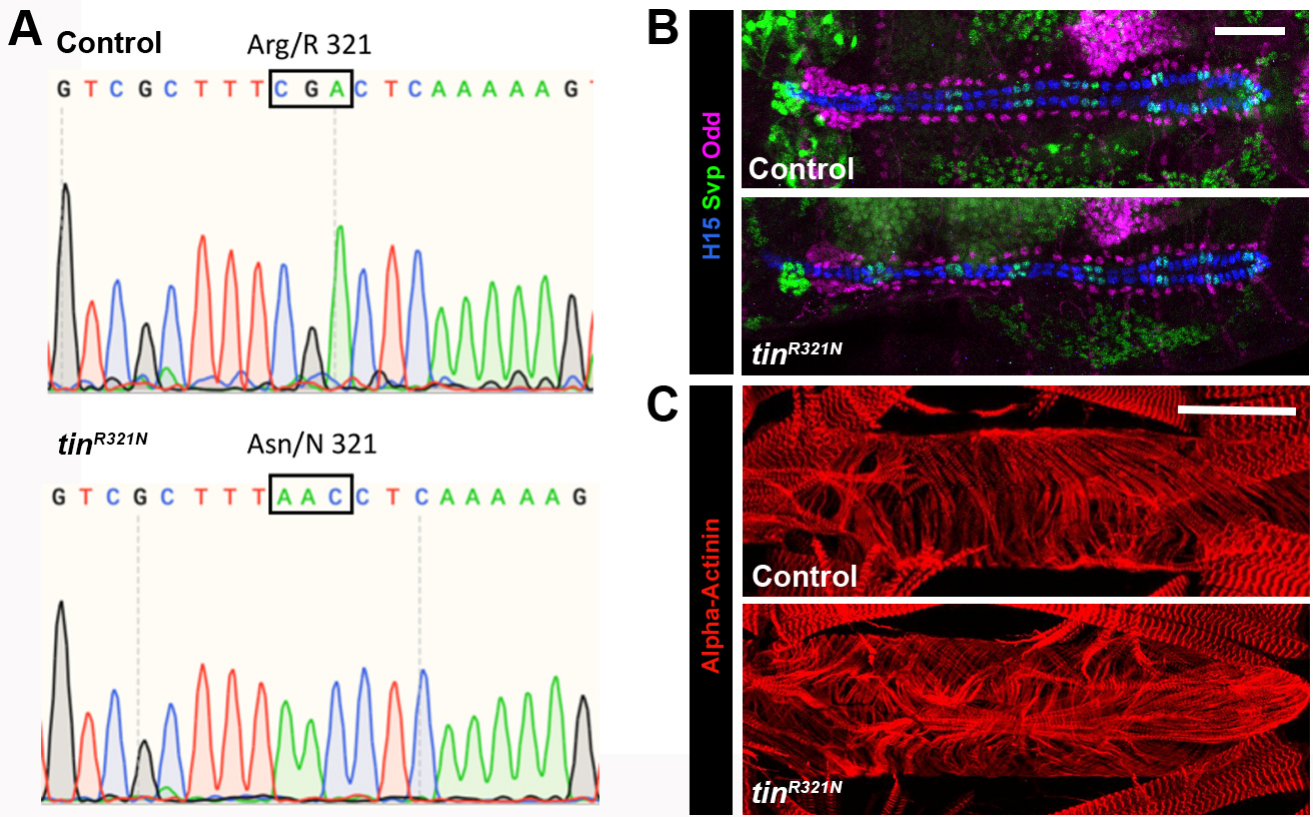
**Generation and analysis of a *tin^R321N^* allele *in vivo*.** (A) Sequence reads of WT *tin* (top) and the *tin^R321N^* allele (bottom), from PCR products generated from homozygous adults. The Arg codon (5′-CGA) is altered to Asn (5′-AAC), and no other changes to the *tin* coding sequence were observed. (B) There were no observable changes in heart cell patterning of *tin^R321N^* homozygotes compared to *vasa-Cas9* controls. Cardiac cells are labeled with anti-H15 (blue) and anti-Svp (green). A subset of pericardial cells is labeled with anti-Odd (red). *n*≥10 embryos for each genotype. (C) Larval hearts from the A6 abdominal segment of *vas-Cas9* (control) and *tin^R321N^* homozygous larvae, labeled with anti-Alpha-actinin (also known as Actn; red) to visualize cardiac myofibrils. No consistent differences in heart structure were observed between control and mutant (representative images shown, see text for quantification). Scale bars: 50 µm.

We next sought to determine whether this mutation affects Tin function in the context of the intact animal. We used CRISPR/Cas9 genome editing to generate a 5′-CGA to 5′-AAC change at codon 321, and isolated one line that showed this alteration with no other changes to the *tin* coding sequence (Fig. 4A). Interestingly, these flies were homozygous viable and fertile, and could be maintained as an homozygous stock.

To determine whether the R321N mutation affected Tin function *in vivo*, we assessed heart formation in control and homozygous-mutant embryos. In WT animals, the mature embryonic heart at stage 16 comprises two parallel rows of cardiac cells forming a cardiac tube, flanked by a number of pericardial cells. Within these two cell populations, there is significant molecular diversity: the mature cardiac cells express either *tin* or the orphan nuclear receptor gene *seven up* (*svp*) (Lo and Frasch, 2001), and the pericardial cells express a number of different transcription factors, with a subset of the cells expressing the C2H2 zinc finger transcription factor gene *odd skipped* (*odd*) (Ward and Skeath, 2000) (Fig. 4B, top). In *tin^R321N^* homozygotes, we did not observe any consistent differences in cardiac and pericardial cell patterning (Fig. 4B, bottom), suggesting that any effect of the point mutation upon *tin* function was not sufficient to affect heart cell specification or patterning.

We next determined whether the point mutation of *tin* affected cardiac growth. During larval development, the cardiac tube generated in the embryo increases significantly in size and then, during pupal development, metamorphoses into the adult heart (Molina and Cripps, 2001). When we studied the heart of fully grown third-instar larvae, we did not observe any differences in organization of the myofibrils between control and *tin^R321N^* homozygotes (Fig. 4C), and there were no differences in heart diameter: heart diameter in segment A7 was 92 µm in *w^1118^* controls and 84 µm in *tin^R321N^* homozygotes (*n*=9 and *n*=8, respectively, not significant in an unpaired two-tailed Student's *t*-test).

**Fig. 5.**
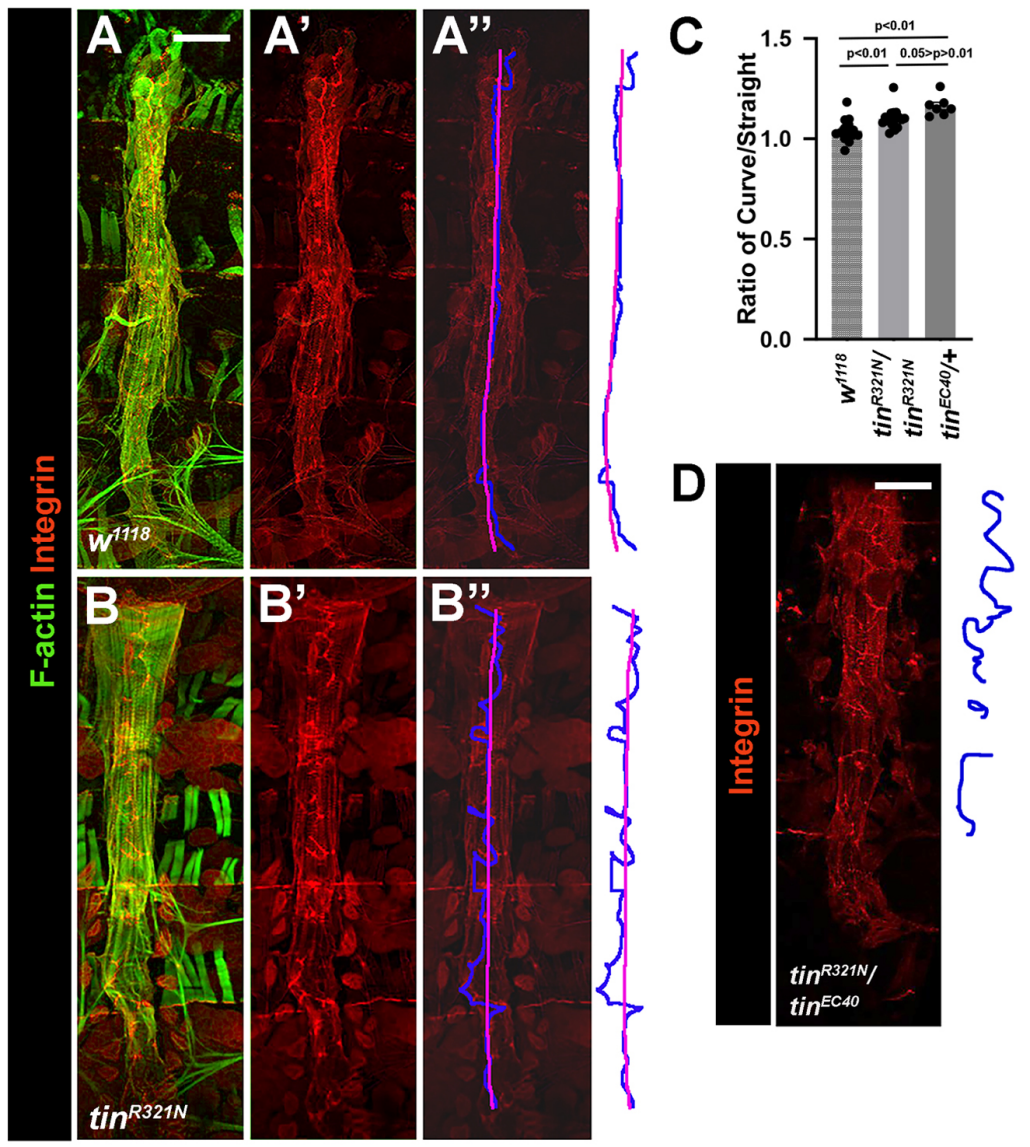
***tin^R321N^* adults have defects in heart cell patterning.** (A-B″) Whole-mount preparations of *w^1118^* (control; A) and *tin^R321N^* homozygous (B) adult hearts, stained for accumulation of F-actin (also known as Act5C; green) and β-Integrin (red). Although the overall structure of the heart did not differ between genotypes based upon the F-actin accumulation, accumulation of Integrin was irregular in the mutants (B′) compared to controls (A′). In controls, a single prominent line of Integrin stain was observed along the ventral midline of the heart, traced by a blue line in A″. This line of stain was irregular in the *tin^R321N^* homozygotes (B″). To quantify the irregular nature of the line, the length of the blue line was calculated relative to that of a single straight line along the ventral midline (magenta in A″ and B″), to give a ratio of curve/straight. (C) The calculated ratio for *w^1118^* (*n*=15) was significantly less than that for *tin^R321N^* (*n*=17), indicating that the line was more irregular in the mutants than in the controls. This phenotype was stronger in *tin^EC40^/+* heterozygotes (*n*=7). Error bars represent s.e.m. (D) Combining *tin^R321N^* with *tin^EC40^* to create *tin^R321N^/tin^EC40^ trans*-heterozygotes resulted in severe disruptions to heart cell patterning, in which the ventral line of Integrin stain was highly disrupted and could not be consistently traced as a single line from anterior to posterior. Scale bars: 50 µm.

Because the heart undergoes remodeling and further growth during the pupal stage, we next analyzed the hearts of adult animals for any defects. Once again, there were no gross defects in heart patterning in the mutants, and heart diameter was unaffected in mutant compared to controls animals. Heart diameter in segment A3 was 59 µm in *w^1118^* controls, 64 µm in *tin^R321N^* homozygotes, 69 µm in *tin^EC40^/+* adults and 55 µm in tin*^R321N^/tin^EC40^* adults (*n*=9, *n*=9, *n*=9 and *n*=7, respectively, not significant in one-way ANOVA). When we studied adult heart structure in more detail, we observed subtle but reproducible defects in the arrangement of the adult heart cells in mutant animals. In WT, the persistent larval cardiac tube increases in size and undergoes extensive myofibrillogenesis to form a muscular tube (Molina and Cripps, 2001). Along the ventral side of this tube are arrayed a series of longitudinal muscle fibers (Fig. 5A). The arrangements of individual heart tube cells can be visualized through staining for accumulation of β-Integrin (also known as Mys), which is enriched at the junctions of each cardiac cell and is particularly visible along the ventral midline of the tube. In WT animals, this line of Integrin staining was relatively straight (Fig. 5A), indicating that contralateral cells meet at approximately the same midline location. However, in *tin^R321N^* homozygotes, the line was far more irregular; in all 17 samples analyzed, this line was continuous but irregular and frequently deviated significantly from the midline (Fig. 5B).

To quantify this phenotype, the ‘length’ of the Integrin line in pixels was calculated using online software (see Materials and Methods). The length of the Integrin line was then compared to a best-fit line representing the least distance along the ventral midline of the heart between the start and stop point of the integrin line (Fig. 5A″) to generate a curve/straight ratio. A perfectly straight line of Integrin stain would score a ratio of 1.0, whereas a line that deviated from the midline would show a ratio greater than 1.0. We calculated these ratios for control and *tin^R321N^* homozygotes, and we observed that the mutant ratio was significantly greater than that for the control (Fig. 5C, *P*<0.01). If these defects in Integrin staining arise as a result of reduced *tin* function, heterozygotes for a *tin* null allele might also show the same phenotype. Indeed, when we analyzed *tin^EC40^/+* adults, defects in Integrin patterning were also observed and were more severe than those observed for *tin^R321N^* homozygotes (Fig. 3C).

A further prediction is that the defects arising from the *tin^R321N^* allele should be enhanced when combined with the *tin* null allele. Moreover, this experiment would further test whether the defects we observed here were due to the engineered mutation of *tin* and not due to a second-site mutation on the same chromosome. To achieve this, we crossed *tin^R321N^* adults to heterozygotes for the *tin* null allele *tin^EC40^* (Bodmer, 1993) and analyzed adult heart structure in *tin^R321N^/tin^EC40^* adults. Because the *tin* null allele would provide zero Tin function, we would predict a stronger phenotype in these *trans*-heterozygous animals than in both the *tin^R321N^* homozygotes and the *tin^EC40^/+* heterozygotes.

In the *tin^R321N^/tin^EC40^* adults, we observed severely fragmented Integrin staining in the majority (9/11) of animals (Fig. 5D), which could not be quantified in the manner described above owing to the breakage in the line of staining. The *tin^EC40^/+* adults also showed fragmentation in the Integrin stain, albeit at a lower frequency (9/16 adults) than for the *trans*-heterozygotes (Table 1).

**Table 1.**
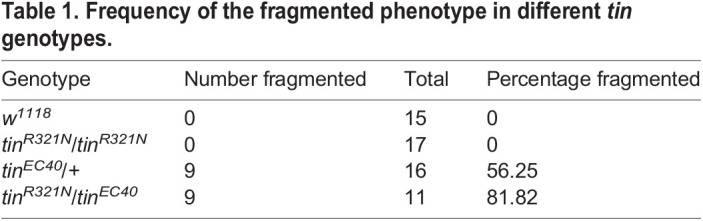
Frequency of the fragmented phenotype in different *tin* genotypes.

Overall, these data demonstrate that the defects in the *tin^R321N^* homozygotes are caused by the genome-edited *tin* allele, because the phenotype is enhanced when combined with a *tin* null allele. Moreover, the *in vivo* studies complement the *in vitro* studies, demonstrating that the Tin^R321N^ isoform is subfunctional, because its phenotype can be enhanced by further reduction in Tin function.

## DISCUSSION

In this study, we tested the feasibility of using *Drosophila* as a model to understand the potential impact of a human variant of unknown significance in the congenital heart disease gene *NKX2.5*. Modeling NKX2.5^K158N^ as Tin^R321N^ generated a polypeptide that *in vitro* showed a strongly reduced ability to interact with DNA and reduced *trans*-activation ability in co-transfection experiments. Because the missense mutation affects the DNA-binding homeodomain of Tin, our findings were consistent with reduced protein-DNA interactions for the mutant isoform. Based upon these effects of the mutation, we anticipated that modeling the same mutation in the endogenous *tin* gene *in vivo* would severely impact heart specification and differentiation. Instead, the *tin^R321N^* homozygotes were viable and fertile, and showed only modest, albeit reproducible, defects in the patterning of cells in the adult heart.

We also demonstrate here, for the first time, a physical interaction between the T-box-encoded protein Doc1 and the cardiogenic factor Tin. Although we only tested the ability of Doc1 to interact with Tin, we feel it is likely that all three Doc paralogs are capable of this interaction based upon their highly similar sequences and expression during heart development (Reim et al., 2003; Reim and Frasch, 2006). A physical interaction between Tin and Doc1 is also consistent with a genetic interaction between *tin* and the three paralogous Doc genes (Reim and Frasch, 2006).

Our studies are also consistent with the documented interaction between mammalian NKX2.5 and TBX5 (Pradhan et al., 2016; Luna-Zurita et al., 2016). Although *Doc1* is not the closest *Drosophila* sequence ortholog of human *TBX5*, the similar roles of Doc proteins in heart specification and the interaction between Doc1 and Tin/NKX2.5 indicate that *Doc1* and *TBX5* are functional orthologs. In support of this, one NKX2.5 residue that lies at the interaction surface between NKX2.5 and TBX5 is K158 (Luna-Zurita et al., 2016), which we show here to also be required for effective interaction of Tin with Doc1. These data suggest that there are common mechanisms underlying the interactions of NK homeodomain proteins with T-box proteins that could represent a mechanistic basis for multiple biological processes. In support of this, the NK homeodomain factor *ceh-51* and the T-box factor *tbx-35* also genetically interact in the formation of the *Caenorhabditis elegans* pharynx (Broitman-Maduro et al., 2009), and several examples exist of homeodomain proteins interacting with T-box proteins (Farin et al., 2007; Nowotschin et al., 2006; Leconte et al., 2004; Boogerd et al., 2008).

At the molecular level, it is interesting to note that the R321N substitution affects both DNA binding and co-factor interaction, and these effects can provide insight into the molecular basis of the mutant phenotype and potential disease mechanisms. Homeodomains are known to fold into three alpha helices, the third of which is the predominant region for interacting with DNA (Gehring et al., 1990; Kissinger et al., 1990). According to the NKX2.5 crystal structure (Pradhan et al., 2012), K158 is a surface-facing residue that lies at the C-terminal end of helix 1. Given the strong homology in homeodomain structure, R321 in Tin likely occupies the same structural location. Such a position on the ‘surface’ of the homeodomain is consistent with a role in co-factor interaction with Doc, as we have confirmed here and as shown by Luna-Zurita et al. (2016). However, how this mutation impacts DNA binding is less obvious. We note that the nearby Y162 residue in NKX2.5 (Y325 in Tin) contacts DNA (Pradhan et al., 2012), so it is possible that the R321N substitution affects the ability of Y325 to connect properly with the DNA-binding site. Alternatively, the R321 residue, given its location at the end of the first alpha helix, might de-stabilize the structure of the helix, causing reduced interaction of the homeodomain with DNA *in vitro*.

Our studies also raise a number of important considerations when the goal is to understand the potential clinical impact of uncharacterized variants. First, there is a clear difference in the severity of the phenotypes when comparing assays performed *in vitro* versus *in vivo*. Based upon the DNA-binding and co-transfection studies, Tin^R321N^ is clearly deficient compared to WT Tin, yet *tin^R321N^* homozygotes show normal heart specification and early differentiation. Thus, the functional deficiencies that were observed *in vitro* appear to be muted *in vivo*. This could be due to different biochemical environments or to the presence of co-factors that can stabilize some deficiencies in Tin function. Our results underline the important contributions that *in vivo* studies make to understanding the phenotypic severity for a given mutant allele.

Second, we show that there is an *in vivo* impact of the *tin^R321N^* mutation and suggest strongly that, in humans, the K158N variant is pathogenic. The observation that the phenotypes do not arise in flies until later in development, when the heart has undergone its maximum period of growth during metamorphosis, suggests that, in humans, this variant might impact later stages of heart development.

Third, we note that the effects of the mutation upon adult flies are quite modest and only exacerbated when heterozygous with a *tin* null allele. These observations underline the importance of accurate phenotypic analyses, which must be reproducible and quantifiable to confirm that subtle differences in heart development or function have occurred. Several groups have pioneered such assays in *Drosophila* that can be brought to bear on this issue (Cammarato et al., 2015; Huang et al., 2022).

Overall, this study demonstrates the feasibility of modeling human genetic variants in *Drosophila* and opens a pathway for analysis of further variants of unknown significance using the *Drosophila* heart model.

## MATERIALS AND METHODS

### *Drosophila* stocks and crosses

*Drosophila* were maintained at 25°C on Jazz Mix medium (Genesee Scientific) for all experiments. Genetic nomenclature is as described at FlyBase.org (Gramates et al., 2022). Embryos used for CRISPR were from flies carrying an X-linked *vas-Cas9* construct obtained from the Bloomington *Drosophila* Stock Center (#51323).

### CRISPR/Cas9 genome editing

CRISPR/Cas9 genome editing was used to generate an R321N substitution in the endogenous *tin* gene. sgRNAs targeting *tin* close to codon R321 (protospacer sequence 5′-CGACTCAAAAAGTATCTGAC-3′) and separately targeting *ebony* (5′-GCCACAATTGTCGATCGTCA-3′, used as a co-CRISPR target; Kane et al., 2017) were ordered from Horizon Discovery Biosciences. A single-stranded donor DNA, comprising the intended nucleotide substitutions plus 50 nucleotides on each side, was also ordered from Horizon Discovery Biosciences. These reagents were sent to Rainbow Transgenic Flies Inc. for injection. G0 adults were crossed to a multiple balancer line containing *TM2* and *TM6* on the third chromosome; each balancer carries a recessive *ebony* mutant allele. G1 flies showing an *ebony* phenotype must therefore have received a mutant *ebony* allele from the G0 parent arising through CRISPR mutation, and were therefore considered as most likely to also carry an engineered *tin* allele. These flies were backcrossed to *TM3, Sb lacZ/tin^EC40^* to test for lethality with the null *tin* allele, and to generate a stable stock of TM3/tin* flies (where ‘*’ indicates a potential genome-edited allele). Genomic DNA from heterozygous or homozygous progeny was subjected to PCR and DNA sequencing to detect the presence of the intended point mutation. This mutant line is available from the authors upon request.

### Protein purification

The Tin homeodomain (HD; codons 301-360) was cloned into the pMAL vector, containing an N-terminal MBP tag, using 5′-NdeI and 3′-XhoI restriction sites. The R321N substitution was introduced using site-directed mutagenesis PCR. The Doc1 coding sequence was cloned into the pGEX vector, containing an N-terminal GST tag, using 5′-BamHI and 3′-XhoI restrictions sites. All constructs were sequence confirmed prior to protein expression.

All proteins were expressed in BL21(DE3) *Escherichia coli* under induction by isopropyl β-d-1-thiogalactopyranoside (IPTG) and grown in standard Luria-Bertani broth supplemented with 100 μg/ml ampicillin. Transformed cells were grown at 37°C to an optical density at a wavelength of 600 nm (OD_600_) of ∼0.6 and induced with 0.2 mM IPTG overnight at 18°C. Cells were harvested by centrifugation (5000 ***g*** for 10 min), and bacterial pellets were resuspended in lysis buffer and flash frozen in liquid nitrogen. Cells were lysed using a Branson digital sonifier and clarified by centrifugation (12,000 ***g*** for 30 min).

For MBP-tagged Tin, cells were lysed in lysis buffer [50 mM Tris-HCl pH 8.0, 500 mM NaCl, 2 mM dithiothreitol (DTT)] and coupled to amylose resin for 3 h at 4°C. Following extensive washing, proteins were eluted with elution buffer (50 mM Tris-HCl pH8, 30 mM NaCl, 2 mM DTT, 50 mM maltose). Final purification was carried out using an S200-Sephadex size exclusion column (GE Healthcare) equilibrated in storage buffer (20 mM Tris-HCl pH 8.0, 200 mM NaCl, 2 mM DTT). Protein was concentrated, flash frozen and stored at −80°C until subsequent use. For GST-Doc1, cells were lysed in lysis buffer as above, and clarified lysate was flash frozen and stored at −80°C until subsequent use.

### Protein methods

Electrophoretic mobility shift assays were performed using standard procedures. The DNA-binding buffer contained 40 mM KCl, 15 mM HEPES pH 7.9, 1 mM EDTA, 0.5 mM DTT, 5% glycerol and 4 µg Poly-dIdC. The binding site was the Tin1 sequence from the *Mp* gene (Lovato et al., 2023 preprint), sequence 5′-GGACTCTTTGGCTGAAGTGTCCGGTGAAA-3′, ordered from Sigma-Aldrich (Tin consensus binding sequence underlined). Equal quantities (1.5 µg) of purified WT Tin-HD and Tin^R321N^-HD were used in each reaction.

Direct Doc1-Tin binding was assessed using standard GST pulldown assays. First, equivalent amounts of GST-Doc1 were coupled to glutathione agarose resin in separate reactions for 30 min at room temperature and extensively washed with PBS to removed unbound protein. Protein-bound resin was then resuspended in wash buffer (20 mM Tris-HCl pH8, 150 mM NaCl, 1 mM DTT, 0.2% Triton X-100) and incubated in the absence or presence of Tin (WT or R321N). GST alone was used as a negative control. For saturation binding experiments (see Fig. 3), Tin proteins were added at increasing concentrations between 0 and 500 nM in respective reactions. Reactions were then washed four times with wash buffer. Following the final wash, samples were resuspended in reducing Laemmli buffer and heated at 80°C prior to being resolved by sodium dodecyl sulfate-polyacrylamide gel electrophoresis (SDS-PAGE). Owing to the similar molecular masses of GST-Doc1 and MBP-Tin, MBP-Tin was detected by western blotting using a rabbit anti-MBP antibody (1:1000; GeneTex, GTX77412) and visualized using a Bio-Rad ChemiDoc imaging system. After detection, blots were stained with Ponceau Red (Thermo Fisher Scientific, J60744) to visualize GST-Doc1 and ensure equivalent loading and transfer. Quantification of bound MBP-Tin was performed using ImageJ, and the saturation binding curve was generated using a one-site specific binding model in GraphPad Prism.

### Tissue culture

Co-transfection assays were carried out essentially as described by Kelly Tanaka et al. (2008). The WT *tin* expression plasmid was pPAc-*tin* (Lovato et al., 2015). The *tin^R321N^* mutant expression plasmid was generated using a Q5 site-directed mutagenesis kit (New England Biolabs). The reporter plasmid was the Mp3D cardiac enhancer from the *Mp* gene (Lovato et al., 2023 preprint) fused to *lacZ* in the parent vector pDONR lacZ attB (Bryantsev et al., 2012). Reporter expression was assessed using a mammalian β-galactosidase assay kit (Pierce Technology, Thermo Fisher Scientific). Reporter expression in the presence of transcription factors was normalized to reporter expression in the presence of empty expression vector. Percentage activation is defined as the level of reporter activity in the presence of the activator (WT Tin or Tin^R321N^), divided by the level of reporter activity in the absence of an activator (set to 100%). Ten replicates were carried out, and the reporter activities were analyzed using one-way ANOVA and post-hoc Tukey tests.

### Generation of anti-Tin antiserum

The coding sequence of *tin* was cloned into the pEXP1-DEST vector (Thermo Fisher Scientific, V96001) utilizing Gateway Technology (Thermo Fisher Scientific, 12536017). A positive clone was transformed into BL21 (DE3) competent cells (Thermo Fisher Scientific, C600003), which was induced for protein purification using Talon Metal Affinity Resin (Takara, 635501). Two New Zealand white rabbits were injected at ten sites with 500 µl Freund's Complete Adjuvant (Sigma-Aldrich, F5881) containing 500 µg purified Tin protein. A small blood draw was carried out 4 weeks later to check progress, and a booster injection of 100 µg Tin protein in 200 µl Freund's Incomplete Adjuvant (Sigma-Aldrich, 344291) was carried out at four sites. Two weeks later, 50 ml blood was drawn to check titer, after which a second booster was administered a week later. Four weeks later, the final blood draw was obtained. Serum was collected by allowing the blood to coagulate at room temperature for 30 min and then centrifuged at 2000 ***g***. The supernatant was transferred to a clean tube and analyzed by immunohistochemistry using a concentration of 1:1000. Approximately 50 ml blood was collected for each blood draw. The protocol was institutional animal care and use committee (IACUC) approved (protocol number 18-200741-MC) prior to commencing the work. Specificity of the antibody was confirmed by staining *Drosophila* embryos, which showed the published pattern of *tin* expression.

### Immunofluorescence and microscopy

*Drosophila* embryos were harvested, fixed and stained as described by Patel (1994). Primary antibodies were mouse anti-Svp [1:100; Developmental Studies Hybridoma Bank (DSHB)], rabbit anti-Odd (1:1000; provided by Dr James Skeath, Washington University in St Louis, St Louis, MO, USA) and guinea pig anti-H15 (also known as Nmr1; 1:1000; provided by Dr James Skeath). At least ten embryos of each genotype were analyzed, and representative images are shown.

Preparation and staining of larval pelts and of adult abdomens to visualize the heart was performed as described by Molina and Cripps (2001). The primary antibodies used were anti-β-Integrin (1:25; DSHB, CF.6G11) and anti-Alpha-actinin (1:2000; DSHB, BB8/384.1). The secondary antibody was Alexa Fluor 555 goat anti-mouse (1:2000; Thermo Fisher Scientific, A-21422), combined with Alexa Fluor 488-Phalloidin (1:500; Thermo Fisher Scientific, A12379). At least five animals of each genotype were analyzed, and representative images are shown.

All samples were imaged using an Olympus FluoView 3000 confocal microscope. Images were assembled in Adobe Photoshop, and any changes to brightness, levels and contrast were applied equally to control and mutant samples.

To quantify the irregularity in Integrin stain for control and mutant samples, confocal images were imported into ImageJ. To measure the length of Integrin stain, for each sample, a freehand line was drawn over the Integrin line. This was masked and then skeletonized, which enabled the determination of the length of the skeleton in pixels. In parallel, a separate line was drawn to best fit the ventral midline of the heart. The irregularity of the Integrin stain was calculated by dividing the length of the Integrin line (in pixels) by the length of the best-fit line (in pixels) to create the ratio of curve/straight as shown in Fig. 5C. At least seven samples were analyzed in this way for each genotype. Control and mutant samples were compared using a two-tailed, unpaired Welch's *t*-test in GraphPad Prism.
